# Wishes and needs of older persons who have experienced a fall: A qualitative study

**DOI:** 10.1002/nop2.303

**Published:** 2019-06-11

**Authors:** Ann Sophia Bertelsen, Jesper Ryg, Tahir Masud, Dorthe S. Nielsen

**Affiliations:** ^1^ Department of Geriatric Medicine Odense University Hospital Odense Denmark; ^2^ Department of Clinical Research University of Southern Denmark Odense Denmark; ^3^ Department of Geriatric Medicine Nottingham University Hospitals Trust NHS Nottingham UK; ^4^ Center of Global Health University of Southern Denmark Odense Denmark

**Keywords:** falling, hermeneutic phenomenology, older fallers, patient perspectives, qualitative study, wishes and needs

## Abstract

**Aim:**

The aim of this study was to explore the needs and wishes in everyday life of older persons who have experienced a fall.

**Design:**

A hermeneutic‐phenomenological design with semi‐structured interviews.

**Method:**

Interviews were analysed using systematic text condensation.

**Results:**

Nine patients (5 women, 4 men) were included. The participants were between 67–95 years old. Seven of the nine participants had suffered from recurrent falls. The analysis revealed four themes: “To maintain meaningfulness in everyday life after experiencing a fall”, “Contact with professionals can be a prerequisite for feeling well and motivated after a fall”, “A need for assistive technology and aids in everyday life” and “Asking for help can be a challenge”. Our findings highlight that older‐person‐centred care and treatment are essential to address the complex variations in needs and wishes of older persons who have experienced a fall.

## 1 INTRODUCTION

Older persons experiencing a fall in their everyday life institute a common problem and a serious challenge for healthcare systems. One‐third of the population over 65 years of age fall at least once a year, and the proportion rises to 50% in older people above 80 years of age (Blake et al., 1988; O'Loughlin, Robitaille, Boivin, & Suissa, 1993). Furthermore, having fallen once increases the risk of falling again (Tinetti, Speechley, & Ginter, 1988). The World Health Organization reports that the number of persons over 60 years is growing faster than any other age group (WHO, 2007). This increase in the proportion of older people at risk is expected to lead to an increase in the number of falls, which makes falling and the associated consequences increasingly problematic worldwide in terms of morbidity and significant health and social care costs for all European Countries in the future (Todd, 2004).

The international literature shows a plethora of information on interventions to try and prevent older people from falling; however, work is still needed to identify how to increase patient concordance with interventions (recruitment, uptake, acceptability, motivation and adherence) to reduce the risk of falls (Todd, 2004).

Further research to close this knowledge gap is therefore important to maximize health professionals' ability to help older persons who have experienced a fall. Furthermore, interventions and services for fallers can only be effective if they appeal to the older age group and meet their wishes and needs. This requires knowledge from a patient perspective in a way that makes it possible for the health professionals to understand what is important in everyday life of an older person after a fall event.

Previous studies have investigated various aspects regarding older persons after a fall event showing that they fear the consequences of falling (Tischler & Hobson, 2005), that falls can result in a decline in health status, ability to undertake activities of living, lifestyle and quality of life (Roe et al., 2009), but also that some accept fear of falling as a part of life (Mahler & Sarvimaki, 2012) and that older people can be embarrassed about wearing visible falls detection technology (Charlton, Murray, & Kumar, 2018). Furthermore, it has been reported that older women make a range of postfall responses and decisions, including engaging in the extra work required to get back to normal, purposefully avoiding people, objects and places, being proactive and planning ahead and putting the fall out of mind (Bergeron, Friedman, Messias, Spencer, & Miller 2016). Finally, indications from prior reports state that health professionals frequently fail to refer people to fall prevention interventions (Dickinson et al., 2011) and that future research could involve older people in decision‐making about falls prevention (McInnes, Seers, & Tutton, 2011).

However, despite these findings, there is still a gap in the existing literature. Further research needs to go beyond exploring how falling has an impact on everyday life and investigate *what* older persons wish and need in their everyday life after experiencing a fall. This knowledge could help health professionals to plan future interventions and services which would appeal to older persons after a fall event. Therefore, the aim of this study was to explore the needs and wishes in everyday life of older persons who have experienced a fall.

## 2 METHOD

The study is reported using the consolidated criteria for reporting qualitative studies (COREQ; Tong, Sainsbury, & Craig, 2007).

### 2.1 Theoretical framework and study design

A hermeneutic‐phenomenological approach was used together with a qualitative explorative design. Semi‐structured interviews were used to gather data to identify the variations in perspectives, thoughts and experiences regarding older persons needs and wishes in their everyday life after experiencing a fall (Vallgårda & Koch, 2011).

### 2.2 Participants and setting

Purposive sampling (Maxwell, 2013) was used to include participants, and 10 older persons (five men and five women) above 65 years of age were invited to participate in the study. One person resigned from participation. The recruitment was conducted at the Falls Clinic at Department of Geriatric Medicine, Odense University Hospital, Denmark between 9 September 2017 and 6 December 2017. The nurses at the Falls Clinic acted as gatekeepers for getting in contact with the participants. We included persons who were cognitively well‐functioning (defined as Mini‐Mental State Examination above 24 points; Folstein, Folstein, & McHugh, 1975), were living in their own home and were able to speak and understand Danish. All participants had experienced a fall within the last 3 months, and none had suffered any fractures.

### 2.3 Data collection

The semi‐structured interviews were conducted using an interview guide containing six guiding questions, which was developed through literature search, experiences from clinical practice and discussion among authors (Table 1). Each interview started with an open question such as “Would you please tell me about your everyday life” to create a relaxed atmosphere and encourage the participants to give an answer using their own words and knowledge. The main author ASB was the interviewer in all the interviews which were held within a week after the participants were asked to participate.

**Table 1:** Interview guide

Guiding questions
1. I would like to hear about your everyday life. Could you describe what your days are like? What do you like to do in your daily life?
2. Can you describe your last fall and how it felt? Have you fallen more than once?
3. Can you describe how your daily life has been after you experienced your fall? Activities, physical state, mental state, mood, social activities, and personal care
4. How do you feel about your fall now? Do you have thoughts about falling again?
5. Is there anything you miss or need in your everyday life?
6. If anything were possible, what would you wish for in your daily life?

The interviews took place in the participants' own home, which was decided by all participants and supported by the research team. The reason for this was to make sure that the participant was in a well‐known environment, as well as eliminating the possible problems with transportation for the participant. In four out of nine interviews, a relative passively participated and sat next to the participant. By including the family member into the interview setting, a trusting environment was created in the interview situation, which helped the participant to share stories, experiences and thoughts.

To validate data from the interviews, the interviewer used the method of reflective listening (Rollnick, Miller, & Butler, 2008), a method where the interviewer repeats the key elements of what the participant has expressed, to secure that all important information is gathered in the right way and thoroughly understood.

### 2.4 Analysis

The transcribed interviews were analysed by the method *systematic text condensation* (Malterud, 2012). This method consists of the following steps: (a) total impression—from chaos to themes. In the first steps of the analysis, a phenomenological approach was used. The aim was to get a general overview of the whole material. Thus, all interview transcripts were read several times; (b) identifying and sorting meaning units—from themes to codes. In the second step, we identified and sorted out meaning units to identify the variation in perspectives and elicit accurate quotes describing the participants' perspectives regarding their needs and wishes in their everyday life; (c) condensation—from code to meaning. In step three, the meaning units were reread several times and the content was reduced into a condensate—an artificial quotation maintaining, which as far as possible has the original terminology applied by the participants; and (d) synthesizing—from condensation to descriptions and concepts. In the fourth step of the analysis, a hermeneutical approach was added to the analysis (Vallgårda & Koch, 2011). In this final step, the aim was to understand and interpret the condensations and synthesize the contents of the condensates into the final descriptions regarding the participants' experiences and perspectives regarding their needs and wishes.

All coding and analysis were performed systematically using the software QSR NVIVO Pro 11. Table 2 shows a schematic example of the steps in the analysis process from meaning unit to condensation, description and theme title.

**Table 2:** Schematic example of the steps in the analysis process from meaning units to condensation, to description and theme title

Meaning units	Condensation	Description	Theme title
“I have no wishes” (AF) “I do not miss anything at all.. ().. Overall, I'm not really displeased with anything” (DF) “It was the fall clinic that called me. I would never have gone to the hospital myself” (CM) “Help is the last thing I'll ask for. As long as I can handle it without help, no one will be called. I have both wallpapered and laid floors here in the house” (BM) ‘I'm annoyed that I had such an experience and had to stay for a day and a half in the hospital. They tear in me and ask if I have pain. No, I have no pain! [...] I don´t care that I´ve fallen’ (BM)	I have no wishes I do not miss anything at all. I'm not displeased with anything The fall clinic called me. I would never have gone to the hospital myself Help is the last thing I'll ask for. I have done everything by myself in this house I'm annoyed that I had such an experience and I don't care that I've fallen. I do not want to spend time at the hospital	The participants did not express any needs or wishes for their everyday life Some participants rejected any need for help and some felt no need for going to the hospital although they had experienced one or more falls. It was of great important to clarify that they were independent and able to manage themselves	“Asking for help can be a challenge”

### 2.5 Ethical considerations

The Danish Data Protection Agency approved the study in accordance with the Act on Processing of Personal Data No. 17/31527. Approval by an official ethics committee was not required according to Danish law. All participants were informed that participation was voluntary, and they could withdraw from the study at any time without explanation. Informed written and verbal consent was obtained from all participants.

## 3 RESULTS

A total of nine persons were included in the study and completed the interviews. The participants were between 67–95 years old. The interviews lasted approximately 1 hr and were recorded and subsequently transcribed verbatim. The characteristics of the study participants are listed in Table 3. The participants' initials have been altered in this article to avoid identification. As shown in Table 3, the age, gender, civil status and type of accommodation varied among the participants. Eight of the nine participants were recurrent fallers.

**Table 3:** Characteristics of the study participants

ID	Age	Gender	Civil status	Number of children	Type of housing	Number of falls	Relative participating in the interview
AF	76	F	Single	0	Rented accommodation	>2	None
AM	79	M	Married	0	Rented accommodation	>2	Spouse
BF	78	F	Married	2	Real estate	2	Spouse
CF	88	F	Single	0	Rented accommodation	>2	None
DF	95	F	Widow	3	Real estate	>2	None
BM	84	M	Married	2	Real estate	1	Spouse
EF	74	F	Widow	2	Rented accommodation	>2	None
CM	67	M	Divorced	2	Rented accommodation	>2	Girlfriend
DM	77	M	Married	1	Real estate	>2	None

Note

The ID abbreviation is fictional.

Abbreviations: F, female; M, male.

The interviews revealed insight into the participant's perspectives of their wishes and needs for their everyday life after their fall experience. Some needs and wishes were directly linked to the fall experience while other needs and wishes were more connected to everyday life in general. In this way, the study gained knowledge about conditions and needs that had importance to the participants in their conduct of everyday life regardless of the fall experience along with wishes and needs in everyday life regarding their fall.

The results of the analysis were categorized into the following four themes: (I) “To maintain meaningfulness in everyday life after experiencing a fall”; (II) “Contact to professionals can be a prerequisite for feeling good and motivated after a fall”; (III) “A need for assistive technology and aids in everyday life”; and (IV) “Asking for help can be a challenge”. The themes are presented in the following sections to illustrate the participant's perspectives, thoughts and feelings and to clarify what was expressed as important and needed in their everyday life after experiencing a fall.

### 3.1 Theme I: to maintain meaningfulness in everyday life after experiencing a fall

The first theme of the analysis revealed that all participants expressed a general wish for maintaining a meaningful life after experiencing a fall. The specific wishes and needs varied depending on what the individual found most important in life and to what gave meaningfulness in their own everyday life.

After experiencing a fall, all participants expressed the importance of not being dependent on others. It was a great wish to be able to do the things they wanted to do in their daily living, like engaging in creative activities such as knitting. Maintaining cognitive competence and keeping one's owns identity was a prominent element:

I wish that I will be able to do what I like to do (CF)

Most of the men participating in the study expressed the importance of being able to drive their car. Not being able to drive by themselves after their fall experience affected their independence and gave a feeling of lost identity:

It is the most important wish for me; to be able to drive by myself. So I do not have to bring my wife along every time (MB)

The participants also expressed an important need for social contact after their fall experience. To be needed and to need others was expressed as an important part of life. To thrive and maintain meaningfulness in everyday life, most participants uttered a need and wish for social contact and caring from others after their fall experience. Participants not having the option of sharing everyday life with others underlined that they at times could feel lonely:

It's just that once in a while, you can sit down and feel … I mean, you could use another person at the other side of the table to talk to (AM)

Although all the basic needs in everyday life were fulfilled, food, cleaning and laundry were delivered to the house of the participant, living with limited social contact became the most important issue in his everyday life and caused a wish for more social contact and caring from others. Although a person came to clean, another person came to get the dirty laundry and a third person delivered a hot meal daily, this social contact did not bring meaningfulness into everyday life of the participant. Empathetic and authentic care and interest from another human being were needed to fulfil the participants' social needs in his everyday life after he had experienced a fall.

### 3.2 Theme II: contact with professionals can be a prerequisite for feeling well and motivated after a fall

When talking to the participants about their wishes and needs in their everyday life after experiencing a fall, several participants expressed that having contact with professionals was a highlight in their everyday life. To avoid falling again, professional help and care had statistically significant importance to most participants and getting specialist knowledge from clinical professionals was praised among the participants. The fact that being in contact with health professionals who wanted to help them avoid falling again was expressed as a great value to the participants and the professional contact motivated participants to attend the Falls Clinic for fall assessment and to comply with the recommendations:

I'd like to avoid falling and I am attending the Falls Clinic and the fact that something is happening gives me a lot (DM)

Getting in touch with the Falls Clinic and being taking care of by clinical professionals was also expressed as being a big dream coming true:

It's my big dream now that they figure out what it is (what caused her many falls) (CF)

Likewise, participating in physical training with a professional physiotherapist was a highlight in everyday life and the participants experienced pleasure and benefits when attending training sessions. Participating in training was described as generating not only physical benefits, but also social and emotional benefits and was expressed as a “highlight of the week”:

I'm glad to attend training with the physiotherapist because we learn a lot […] I really think it helps. They are skilled […] I'm looking forward to coming there […] that's a highlight of the week (AM)

Thus, receiving professional help and care was of great importance in everyday life of the participants, who had wishes, needs and dreams of getting help to avoid falling again and maintain independency. The participants spoke warmly about the professional caregivers, doctors and therapists and expressed that the given fall interventions gave them the needed information, motivation and guidance. Advice on how to get up from the floor after a fall and information from the clinical nurses about how drop in blood pressure, when rising from a bed, can be associated with risk of falling, was emphasized by most participants as being important and needed information to manage daily living after a fall.

The participants also emphasized that the health professionals at the Falls Clinic had a holistic approach in their way of treating the patients, and this was highly praised among the participants:

They do a great job to get around the whole person (BF)

The participants felt that the health professionals had skills, which embraced the whole person and not just one single part of the body, which was expressed as needed to receive help and care that was in accordance with the daily life of older persons who have experienced a fall.

### 3.3 Theme III: a need for assistive technology and aids in everyday life

The third theme of the analysis illustrated that participants expressed a need for assistive technology and aids in their everyday life after a fall event to feel safe and independent from others. Several participants expressed wishes for several technologies and help supplies that could help them in their everyday life to reduce the risk of falling again, being able to manage daily activities by themselves, like taking a shower and to do everyday activities without being depending on help from others:

I would really like to have a handle inside the shower, one I could hold on to. Because when you rinse the soap out of your hair, you'll lean back with your eyes closed (EF)

The need of assistive technology and aids was based on a wish for preventing another fall as well as a wish for assistance in the event of a fall. Mobile walkers were perceived as being able to reduce the risk of further falls, and an emergency call device was expressed as a lifeline for receiving help if the older person was to experience another fall. By expressing these needs, the participants were planning ahead and wanted to implement safety technologies to help managing and preventing further falls.

The analysis showed that the request for assistive technology and aids was only expressed by female participants. Some male participants expressed that planning ahead and thoughts about fall prevention were just not present in their minds because they did not think about their fall experience in their everyday life.

### 3.4 Theme IV: asking for help can be a challenge

As a fourth theme, the analysis identified a group of participants who had difficulties expressing their wishes and needs in their everyday life after they had experienced a fall and some of these participants directly rejected any need for help in their daily living. This group of participants felt no need to contact health professionals and had no need for going to the hospital although they had experienced one or more falls:

It was the Falls Clinic that called me. I would never have gone to the hospital myself (CM)

These participants did not perceive their fall as a problem, but as a result of either “bad luck” or as an age‐related phenomenon, which was unavoidable. The participants seemed not to be aware of the possibilities of getting professional help, help supplies or treatment to optimize their everyday life.

For some participants, it was of great importance to clarify that they, despite their fall experience, were independent and able to manage the daily life by themselves:

Help is the last thing I'll ask for. As long as I can handle it without help, no one will be called. I have both wallpapered and laid floors here in the house (BM)

Therefore, suddenly having a fall experience and being in need for help was perceived as an annoying element in everyday life, where the participant most of all would do without:

I'm annoyed that I had such an experience and had to stay for a day and a half in the hospital. They tear in me and ask if I have pain. No, I have no pain! [...] I don´t care that I´ve fallen! (BM)

Going from a feeling of being a healthy fit man to the one who is in need of help and is getting in touch with the healthcare system contributed to the feeling of adopting a new identity as an “older frail person”, and thus the need for help was being denied and rejected. Both men and women were present in this group of participants; however, we found most of the men rejecting the need for help and being silent about their needs and wishes after their fall experience.

## 4 DISCUSSION

Our study revealed that the needs and wishes of older persons who have experienced a fall are complex and vary depending on what the older persons define as meaningful in their everyday life. To feel that everyday life is meaningful, older persons after a fall event need to be able to do the things they want to do and to keep on doing the things they have been used to do. Our findings highlight the importance of providing treatment, care and services that contribute to what the individual define as meaningful in their everyday life after experiencing a fall. Healthcare services should help the person to sustain the feeling of being an independent human being doing the things they have been used to do before the fall experience.

The study emphasizes that care and interest from others are great social needs in everyday life of older persons who have experienced a fall and contact with health professionals within a holistic approach are valued and expressed as a highlight in everyday life after a fall event. A need and wish for assistive technology and aids seems furthermore to be represented among women, who tend to plan to avoid another fall. The study also identified an expression of no needs and a denial of need for help which could represent a lack of knowledge about possible treatment opportunities and services or a norm where need for help after a fall event is considered as a threat to the individual identity and independency.

To our knowledge, this is the first study to explore the needs and wishes in everyday life of older persons who have experienced a fall. But our findings contribute to other studies showing that effective health care from a highly competent staff is valued and wished for among older people (Hallgren, Ernsth Bravell, Dahl Aslan, & Josephson, 2015). These findings also contribute to the statements by WHO, outlining that it is highly important that health professionals and caregivers are aware of the great variation in patients wishes and needs and that healthcare services must provide older‐person‐centred care which meets the needs of the individual older person, not only focusing on the specific disease (Beard et al., 2016; WHO, 2018).

Regarding the need and wish for assistive technology, another study likewise reported that medical devices are needed and valued (Schoberer, Breimaier, Mandl, Halfens, & Lohrmann, 2016). Additionally, our analysis reveals that the wish for assistive technology and aids has a tendency to be gender‐dependent since only female participants requested this. Similarly, Bergeron et al. (2016), assessing older women's responses and decisions after a fall, reported that several women were planning ahead and implementing safety measures to help manage and prevent future falls (Bergeron et al., 2016). Correspondingly, a study with both men and women reported how a male participant believed that his pride was a barrier for making contingency plans. The male participant did not like using assisting devices (such as his four‐wheeled walker), which he believed would stigmatize him as someone who needed help (Charlton et al., 2018). These findings therefore indicate that the need for assistive technology and aids could be even higher than shown in our study, but male persons might have refrained in expressing these needs after their fall experience.

In contrast to the group of participants who expressed a great need and wish for getting professional help and assistive technology and aids into their everyday life, our study furthermore identified a group of older fallers, who acted silently and did not express any expectations or needs for professional help after their fall experience. In line with this, McInnes et al. found that a fall was not necessarily categorized as a health problem but more as either a result of “just sheer accidents” or because of inattention rather than to a persistent vulnerability (McInnes et al., 2011). Furthermore, they found that being at risk of falling was perceived as being synonymous with frailty and therefore not relevant to those who did not perceive themselves in that way (McInnes et al., 2011). In this way, the denial of need for treatment and help in our study might reflect a desire to maintain independence and not being willing to make any changes (Pereles, Jackson, Rosenal, & Nixon, 2017). However, this expression of no need for help in everyday life after a fall event could also be explained by a lack of information and knowledge about the possible treatment and prevention services regarding fall experiences. Nevertheless, if the expression of being in no need for help is influenced by either lack of knowledge or is based on a denial of needs, there is room for providing better information in society. Older persons' refusal or non‐uptake of services are not an objective free choice but rather the result of a set of external and potential modifiable circumstances (Howse, Ebrahim, & Gooberman‐Hill, 2004). On the basis of this, we therefore encourage health organizations and municipalities to provide information to older citizens to supply them with sufficient knowledge, so older persons are able to know, judge and express what they wish and need in their everyday life after experiencing a fall. The provided information should contribute in a way that older individuals do not feel a lower self‐esteem because they have fallen and then keep away from expressing needs for help because they do not want a label of a “frail old faller”. It is of utmost importance that fall information and advice is formed and communicated in a way that do not let older persons feel stigmatized (Yardley, Donovan‐Hall, Francis, & Todd, 2006). A way to address this complex topic is to involve older people in service planning (Howse et al., 2004). Our study emphasizes that future research and the healthcare system, in general, should develop targeted initiatives that address older persons who are having difficulties expressing needs and wishes in their everyday life after experiencing a fall.

### 4.1 Limitations

In qualitative research, it is often discussed when data saturation are achieved and in our case with only nine participants this might be relevant. However, due to the uniqueness of each person, it can be argued that no data can be truly saturated (Wray, Markovic, & Manderson, 2007). The fact that eight of the nine participants were recurrent fallers might have influenced the results. However, we did not find any association between the recurrent fallers and their perspectives and answers in the interviews. Further research including a larger sample size might contribute to this element.

Our study was a single centre study performed in Denmark based on the certain context, setting and the conditions of the participants. Therefore, it might not be possible to generalize our results to other older persons. However, our results give new insights that can be transferred to other groups of older persons in similar settings and within countries which are comparable to Denmark. Finally, we excluded residents living in nursing homes, patients not speaking Danish and cognitively disabled persons. In this way, the study does not represent the general outpatient fall population since the study focuses on a limited group of well‐functioning community‐dwelling older persons. However, we aimed for maintaining a homogeneous group of patients with a degree of variability into the sample (in terms of age, gender, civil status and type of accommodation) to get powerful results related to this type of population. To assess possible variations in patient‐reported outcomes, future studies should include the even more vulnerable and frail patient groups. Thus, we recommend that further research should explore the wishes and needs after a fall experience in older persons living in nursing homes, older persons with minority background and older persons who are cognitively disabled.

## 5 CONCLUSION

Data from this study add new information and awareness about older persons who have experienced a fall and clarify expression of their wishes and needs in their everyday life after a fall event. The study showed that needs and wishes of older people who have experienced a fall are complex and vary depending on what they define as meaningful in their everyday life. Care and interest from others are a great need in the everyday life of most older people after a fall event. Need and wish for assistive technology tend to be more frequent among women, whereas contact to professionals are valued and expressed as a highlight in the everyday life of both men and female. The study also pointed out that asking for and receive help can be a challenge and some older persons who have experienced a fall might have barriers to ask for and receive help in their everyday life after their fall event.

## CONFLICT OF INTEREST

The authors have no conflicts of interests.

## AUTHOR CONTRIBUTION

ASB, JR and DN: conception and design of the work. All authors made substantial contribution to the analysis and interpretation of data. ASB and DN: first draft preparation. All authors revised it critically for important intellectual content. All authors approved the final version for publication and agree to be accountable for all aspects of the work.
